# Cost-effectiveness of diagnostic technologies for mycobacterium tuberculosis infection in India and Brazil

**DOI:** 10.1371/journal.pgph.0003638

**Published:** 2024-11-13

**Authors:** Saima Bashir, Shehzad Ali, Seda Yerlikaya, Mary Gaeddert, Lara Goscé, Molebogeng X. Rangaka, Claudia M. Denkinger

**Affiliations:** 1 Department of Infectious Disease and Tropical Medicine, Center of Infectious Diseases, Heidelberg University Hospital, Heidelberg, Germany; 2 Department of Epidemiology and Biostatistics, Schulich School of Medicine and Dentistry, Western University, London, Ontario, Canada; 3 Institute for Global Health, University College London, London, United Kingdom; 4 Department of Infectious Diseases Epidemiology, Faculty of Epidemiology and Population Health, London School of Hygiene and Tropical Medicine, London, United Kingdom; 5 German Center for Infection Research, Partner Site Heidelberg, Heidelberg, Germany; University of Ottawa, CANADA

## Abstract

The economic value of new skin-based tests and blood-based interferon-γ release assays (IGRAs) for tuberculosis (TB) infection is not yet well-established. This study evaluates the cost and cost-effectiveness in two high-burden countries by comparing:(a) new skin-based tests(Diaskintest and Cy-Tb) with the purified protein derivative (PPD)-tuberculin test (TST);(b) IGRAs (Standard E TB-Feron ELISA (TBF))with approved IGRAs (QuantiFERON-TB Gold Plus (QFT-GP)and TSPOT.TB); and (c) the best performing skin-based test with the best performing IGRA) based on cost effectiveness. In this paper, we developed a decision tree model for India and Brazil from a health system perspective. To quantify the effect of parameter variability and uncertainty, we performed both univariate and probabilistic sensitivity analysis. The study findings reveal that among skin-based tests, the Diaskintest is more cost-effective compared to TST-PPD at 22.6 USD and 41.0 USD per correctly diagnosed case of TB infection for Brazil and India, respectively. For blood-based assays, TSPOT.TB outperforms QFT-GP and TBF due to its lower cost and higher effectiveness. When compared with Diaskintest, TSPOT.TB has an incremental cost of approximately 8 USD and 6 USD for India and Brazil respectively but is more effective. The incremental cost-effectiveness ratio (ICER) was 74 USD and 55 USD for India and Brazil, respectively. In summary, while Diaskintest is potentially cost-saving when compared to TSPOT.TB in these two high-burden TB countries but the TSPOT.TB demonstrates higher effectiveness.

## Introduction

About a quarter of the world’s population is estimated to be infected with tuberculosis (TB) [1], which is defined by an immunological response to stimulation by *Mycobacterium tuberculosis*(Mtb) antigen, without any clinically evident active TB disease [2]. As tuberculosis infection (TBI) can progresses to active disease in a subset of people, diagnosis and treatment of TBI constitute an integral component of the World Health Organization (WHO)’s guideline to meet the End TB Strategy’s goals [3]. However, for TBI, there is no gold standard test. Skin-based in-vivo purified protein derivative (PPD)-tuberculin test (TST) and selected blood-based interferon-γ release assays (IGRAs) are recommended by WHO for the diagnosis of TBI [2]. For the TST, PPD, or tuberculin, which is a mixture of species-nonspecific antigens, is injected intradermally. The presence of an Mtb infection is then indicated by the size of in duration resulting from PPD-induced hypersensitivity 48–72 hours after the injection. There are several TST-PPD products available [4]. IGRAs, on the other hand, *are in vitro* tests that measure the cell-mediated immune response to Mtb-specific protein antigens. In 2018, the WHO endorsed QuantiFERON-TB Gold (QFT-G; QIAGEN, Germany),Gold In-Tube (QFT-GIT; QIAGEN, Germany), and TSPOT.TB (Oxford Immunotec, UK) [5]. Despite the fact that both tests TST-PPD and IGRAs are considered to be important tools in TB control, their implementation and scale-up have been limited [6]. For the TST-PPD, this is primarily due to operational challenges, with the test requiring two clinical visits, as well as recurring supply chain challenges [7]. On the other hand, IGRAs show better specificity and require only one visit [6, 8, 9]. However, the cost of the supplies, the need for expensive laboratory infrastructure, and phlebotomy expertise can be prohibitive [6, 7, 10, 11].

New skin tests targeting Mtb-specific antigens (ESAT-6 and CFP-10), similar to IGRAs, have been developed, combining TST’s lower cost with IGRA’s specificity [12, 13]. These include Cy-Tb(Serum Institute of India, Pune, India), Diaskintest (Generium, Moscow, Russia) and EC-skintest (also known as C-TST; Anhui Zhifei Longcom, Hefei, China).Another novel skin test known as the DPPD test (Host Directed Therapeutics Bio Corp, Seattle, WA, USA), on the other hand, uses a recombinant protein called DPPD that is also specific to Mtb [14]. In 2022, the WHO recommended the use of this new class of Mtb antigen-based skin tests, specifically Cy-Tb, Diaskintest, and C-TST, to detect TB infection [13].

Novel IGRA tests have also been developed, commercialized, and reviewed by the WHO. This review included several new tests: QuantiFERON-TB Gold Plus (QFT-Plus; QIAGEN), QIA reach QuantiFERON-TB (QIAreach; QIAGEN), Wantai TB-IGRA (Beijing Wantai Biological Pharmacy Enterprise), Standard E TB-Feron ELISA (TBF, SD Biosensor), and T-SPOT.TB 8 with T-Cell Select (Oxford Immunotec) [15, 16]. Among these, the WHO recommended Wantai TB-IGRA and QFT-Plus based on comparable performance to previously recommended IGRAs. However, the WHO concluded that there was insufficient data available to recommend the other tests at that time [16].

With the introduction of new WHO-recommended IGRAs and skin tests that exhibit similar performance characteristics, factors such as cost and operational complexity will be critical components in driving scale-up. In a recent study, Goscé and colleagues evaluated the cost effectiveness of Diaskintest, TST and IGRA QFT test across Brazil, South Africa and UK [17]. However, there remains a scarcity of evidence regarding the comparative analysis of cost and effectiveness of between these new skin-based tests compared with prior TST as well as the newly developed IGRAs against the previously approved one and against each other. Thus, to inform decision-makers in selecting the most appropriate test, we performed a comparative cost-effectiveness analysis of these new TBI tests and IGRAs in comparison to prior WHO recommended tests and against each other.

## Materials and methods

### Technologies compared in the economic analysis

**Fig 1:**
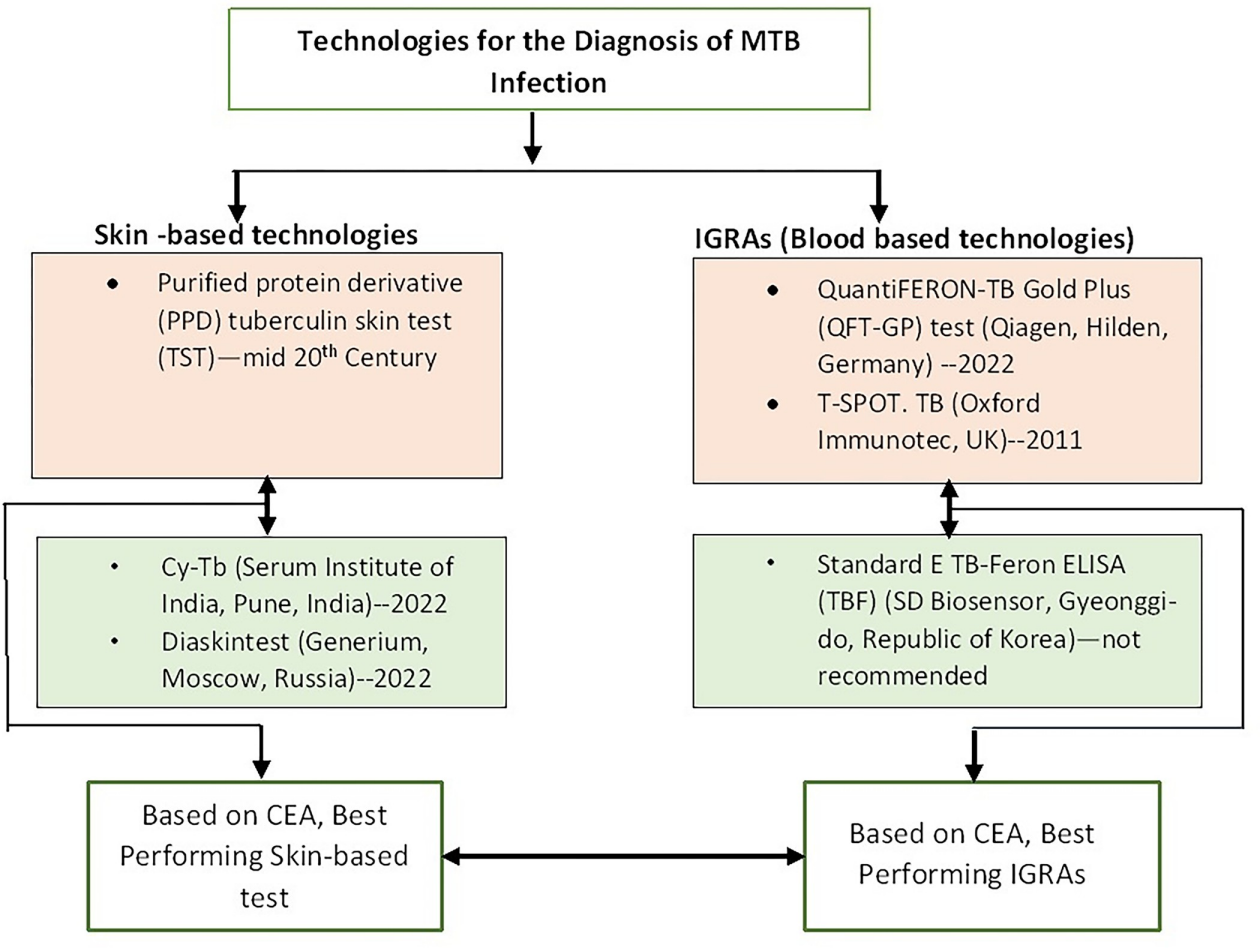
MTB: Mycobacterium Tuberculosis, IGRAs: Interferon-γ Release Assays; CEA: Cost effectiveness analysis, both top boxes (orange) are the tests recommended by WHO; the double pointed arrows show comparison.

This study models the cost-effectiveness of newly developed skin-based tests, namely Cy-Tb and Diaskintest, compared to TST-PPD. In addition, the study evaluates the newly developed IGRA test(TBF) in comparison to WHO endorsed IGRAs (QFT-GP and TSPOT.TB). Due to unavailability of data, Wantai TB IGRA and C-TST were not included in the analysis. Furthermore, based on cost effectiveness, the best performing skin-based test is compared to the best performing IGRA (Fig 1).

### Model and model parameters

We employed a decision analytical model using a decision tree approach to evaluate the cost effectiveness of various diagnostic strategies for TBI in the general population, including the vulnerable groups, immunocompromised patients, and TB contacts. The model is parametrized for two high TB burden countries, India, and Brazil, both of which are included in WHO’s list of the 30 high TB burden countries [18]. We explored whether the uptake of these tests’ impacts TB reduction in countries with diverse regional contexts, population characteristics and costing structures. Model parameters, diagnostic accuracy estimates (sourced from the recent systematic review and meta-analysis) [10, 12, 15, 19], and estimated unit costs are presented in Table 1.

**Table 1:** Model parameters.

	Base-case value	Low value	High value	PSA probability distribution	Source
**Clinical and epidemiological parameters: India**					
Prevalence of TBI	0.391	0.343	0.45	Beta	Global Burden of Disease, 2017
Probability of loss to follow up	0.120	0.030	0.35	Beta	Assumed to be same as Brazil
**Clinical and epidemiological parameters: Brazil**					
Prevalence of TBI	0.256	0.225	0.29	Beta	Global Burden of Disease, 2017
Probability of loss to follow up	0.120	0.030	0.35	Beta	Loureiro et al., 2019
**Test parameters: Skin based tests**					
Sensitivity TST-PPD	0.882	0.782	0.94	Beta	Krutikov et al., 2021
Sensitivity Diaskintest	0.912	0.817	0.96	Beta	Krutikov et al., 2021
Sensitivity Cy-Tb	0.861	0.824	0.89	Beta	Krutikov et al., 2021
Specificity TST-PPD	0.933	0.902	0.95	Beta	Krutikov et al., 2021
Specificity Diaskintest	0.992	0.797	1.00	Beta	Krutikov et al., 2021
Specificity Cy-Tb	0.979	0.940	0.99	Beta	Krutikov et al., 2021
**Test parameters: Blood based tests**					
Sensitivity QFT-GP	0.908	0.800	0.96	Beta	Ortiz-Brizuela et al., 2023
Sensitivity TSPOT.TB	0.909	0.800	0.96	Beta	Ortiz-Brizuela et al., 2023
Sensitivity TBF	0.875	0.732	0.96	Beta	Ortiz-Brizuela et al., 2023
Specificity QFT-GP	0.978	0.955	0.99	Beta	Ortiz-Brizuela et al., 2023
Specificity TSPOT.TB	0.981	0.934	1.00	Beta	Krutikov et al., 2021
Specificity TBF	0.953	0.906	0.98	Beta	Ortiz-Brizuela et al., 2023
**Cost per test: Skin based tests: India**					
Diaskintest	7.86	6.95	8.37	Gamma	Value TB and Goscéet al., 2023
TST-PPD	6.38	4.96	6.88	Gamma	Value TB
Cy-Tb	7.83	7.30	8.36	Gamma	Value TB and Manufacturer
**Cost per test: Blood based tests: India**					
QFT-GP	23.73	16.23	33.02	Gamma	Value TB, and GDF Catalogue
TSPOT.TB	19.98	18.16	21.19	Gamma	Value TB, and GDF Catalogue
TBF	20.84	12.90	28.80	Gamma	Value TB, and Manufacturer (SD Biosensor)
Cost of medical visit	4.45	3.01	5.80	Gamma	Value TB
**Cost per test: Skin based tests: Brazil**					
Diaskintest	5.38	3.79	7.10	Gamma	Goscéet al., 2023
TST-PPD	7.85	5.11	11.07	Gamma	Steffen et al., 2020
Cy-Tb	5.38	4.15	7.09	Gamma	Manufacturer and Steffen et al., 2020
**Cost per test: Blood based tests: Brazil**					
QFT-GP	21.43	12.79	32.45	Gamma	GDF Catalogue, and Steffen et al., 2020
TSPOT.TB	17.68	14.50	19.97	Gamma	GDF Catalogue, and Steffen et al., 2020
TBF	20.84	9.24	27.58	Gamma	Manufacturer (SD Biosensor), and Steffen et al., 2020
Cost of medical visit	6.74	3.37	10.10	Gamma	Steffen et al., 2020

Costs provided (inflated) in 2021 USDs; LTBI = Latent Tuberculosis Infection. IGRA = interferon-γ release assays. QFT GP = QuantiFERON-TB Gold Plus. TST-PPD = tuberculin skin test-purified protein derivative. TBF = Standard TB-feron Elisa; Base-case value is the average value and high and low values are the95% Confidence bounds.

#### Model design

**Fig 2:**
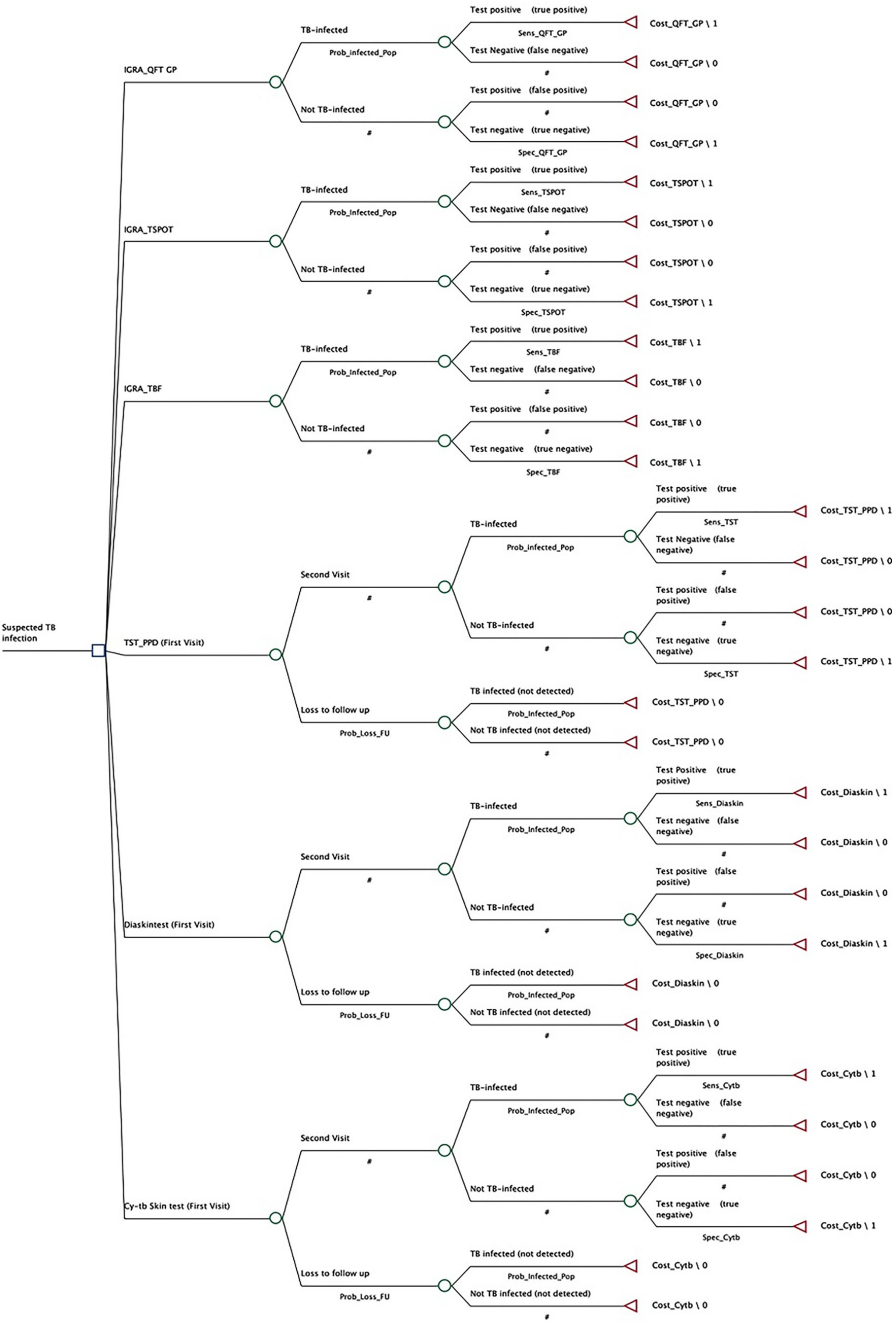
IGRAs: Interferon-γ Release Assays; QFT GP = QuantiFERON-TB Gold Plus. TST-PPD = tuberculin skin test- purified protein derivative. TBF = Standard TB-feron Elisa; Sens and Spec show the sensitivity and specificity of the diagnostic technologies respectively.

The decision tree model is conceptualized by following guidelines of the Professional Society for Health Economics and Outcomes Research [20] and considers a short term time horizon. Two decision trees are constructed for both countries separately using the same structure (Fig 2). The decision trees map the diagnostic pathway of patients, tracking outcomes based on whether they are being infected for TB or not with each diagnostic tool. Specifically, each model includes separate branches for all the diagnostic technologies, with each branch further divided into two pathways: one for TB infected individuals and one for non-infected individuals, based on the TBI prevalence. For skin-based tests, additional branches account for potential loss to follow up prior to the second visit, which is necessary for completing the test. The pathways for both TB infected and non-infected individuals include potential test outcomes: positive and negative results of the tests determined by the sensitivity and specificity of each diagnostic technology.

#### Cost data

All costs incurred at the health-system level are included in the analysis, as the health system typically covers most costs related to the TBI diagnostic tests. However, due to data limitations, patient costs could not be incorporated in the analysis. The cost data has been adjusted to 2021US Dollars(USDs). We conferred with the manufacturers of novel IGRAs and new skin tests to source the unit cost estimates for the testing kit. Unit cost estimates for testing kits were provided by Qiagen (manufacturer of QFT-GP), SD Biosensor, (manufacturer of Standard TBF) and Generium, (the manufacturer of the Diaskintest). For the other tests, cost estimates were obtained from the literature [17, 21, 22]. Additionally, the unit cost per testing kit of QFT-GP and TSPOT. TB was obtained from Stop TB Partnership’s Global Drug Facility (GDF) catalogue.

Costing information for the analysis was sourced from various literature references. For Brazil, this included cost related to human resources (such as nursing staff and lab technician time), consumables (e.g. gloves, syringes with needles, cotton, alcohol, box for syringes, thermic box, ice bag, ruler and thermometer with alarm), and equipment (e.g. ELISA washer and reader, incubator, centrifuge, computer, printers, fridge) [10, 21, 23]. For India, the cost data were extracted from VALUE TB, and it included the cost of building, laboratory and medical equipment, furniture, clinical staff, medical supplies, others (non-medical supplies), capital maintenance, utilities, and other recurrent cost. In VALUE TB, the TST-PPD and Immunocheck TB Platinum kit (Immonoshop, India) are used for the diagnosis of TBI in children [22]. To determine the unit cost of the included tests in the study, the costs listed above (excluding the testing kit cost) were added to the unit costs provided by the manufacturers for the testing kit.

### Effectiveness data

The diagnostic accuracies of the diagnostic tests included in the study are based on recent systematic reviews and meta-analyses [12, 15]. Given the challenge in determining the specificity of Diaskintest from the literature, we assumed it to be equivalent to that of the IGRA (QuantiFERON-T Gold In-Tube)as both tests use the same target antigens [17]. We adopted the number of correct TBI cases detected as an outcome measure. For skin-based tests (Diaskintest and Cy-Tb), a correct case identified is defined using TST-PPD as reference standard, while, for TBF, QFT Gold Plus is used as reference standard. The prevalence of TBI in the general population was extracted from Global Burden of Disease study for both countries [19]. As compared to IGRAs, skin-based tests require two clinical visits for a thorough TBI diagnostic process and for valid results, it’s essential to read them within the suggested timeframe of 48 to 72 hours. There is a likelihood of experiencing loss to follow-up. The probability of loss to follow-up for skin-based tests was derived from existing literature for Brazil [10], and for India, it was assumed to be equivalent due to lack of data.

### Economic analysis

We calculated the incremental cost-effectiveness ratio (ICER) as incremental costs per number of correct TBI cases identified for the following strategies; 1) novel skin tests (Diaskintest and Cy-Tb) compared to TST-PPD, 2) IGRAs (QFT-GP and TSPOT.TB) compared to new blood-based test (TBF), and then 3) best performing skin-based test compared to best performing IGRAs in terms of cost effectiveness using the decision tree (Fig 2). To assess the cost-effectiveness of each diagnostic strategy compared to the standard of care, we related the costs and the effects, measured as number of correct TBI cases identified, to its comparator. Outcomes are expressed separately as ICERs for each comparison [24, 25], calculated using the following formula.


$$ICER=\frac{\left(expected\;unit\;cost\;of\;intervention-expected\;unit\;cost\;of\;comparator\right)}{\left(expected\;unit\;outcome\;of\;intervention-expected\;unit\;outcome\;of\;comparator\right)}$$

### Sensitivity analysis

**Fig 3:**
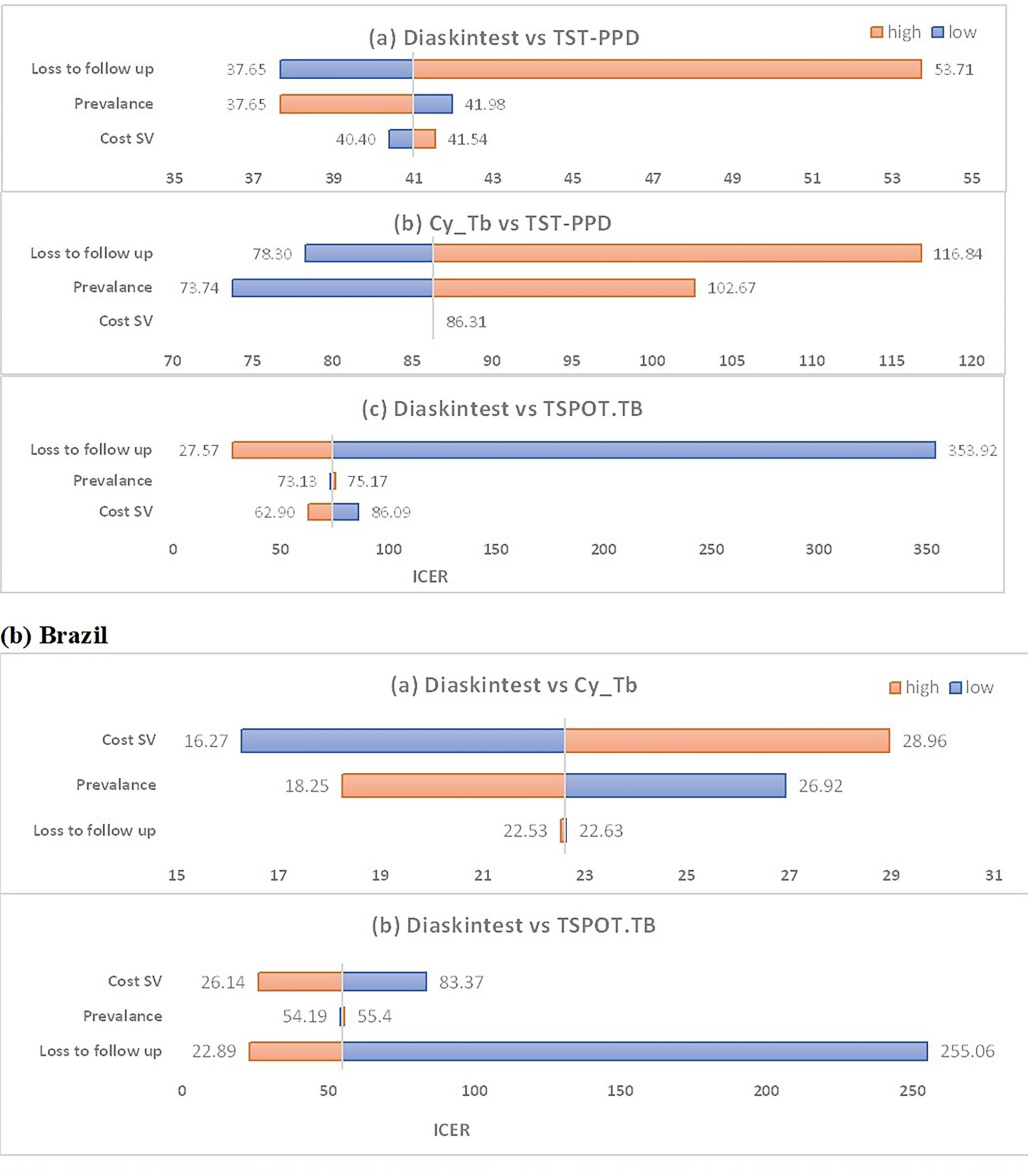
Costs are provided (inflated) in 2021 USDs. ICER = Incremental cost effectiveness ratio, TST-PPD = tuberculin skin test- purified protein derivative, Cost SV Cost of the second medical visit in the skin-based tests. The bar shows the high and low estimates of the average ICER estimates and the values labeled outside the bar are high and low ICER values obtained from using high and low parameters values.

**Fig 4:**
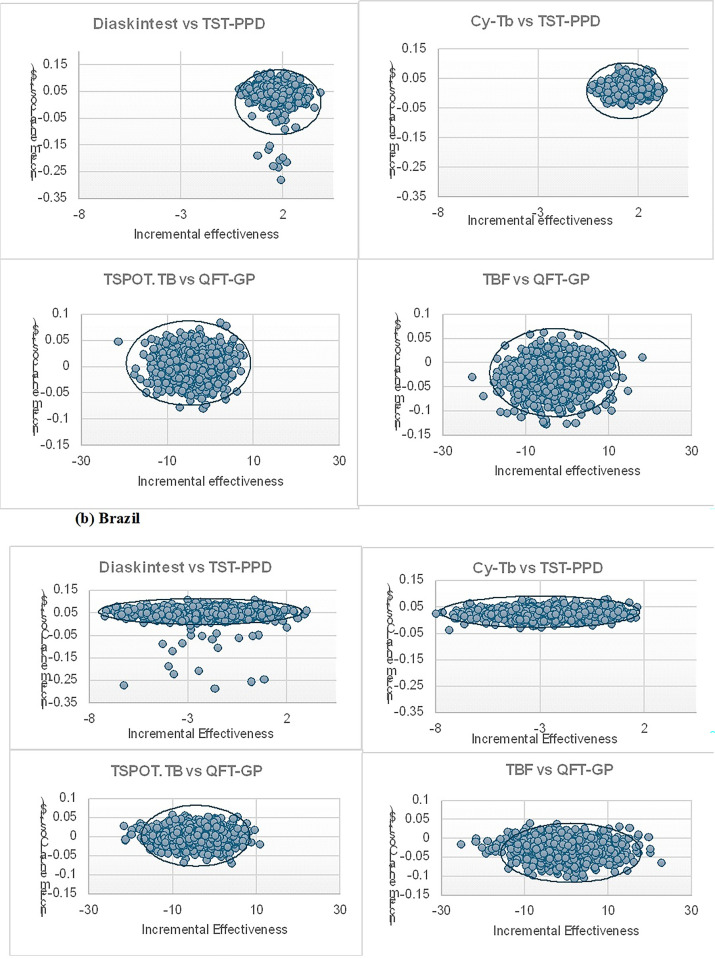
Incremental Cost-effectiveness scatter plot for both countries (a)India and (b) Brazil. The first two scatter plots in each figure show 1000Monte Carlo simulations for the incremental costs (USD, 2021) and incremental effectiveness in correct case diagnosed with that of Diaskintest and Cy-Tb compared with TST-PPD strategy. The second two plots show 1000 Monte Carlo simulations for the incremental costs (USD, 2021) and incremental effectiveness in correct case diagnosed with that of TBF and TSPOT.TB compared with QFT-GP strategy. QFT-GP QuantiFERON TB Gold Plus, TBF TBF = Standard TB-feron Elisa, TST tuberculin skin test.

**Fig 5:**
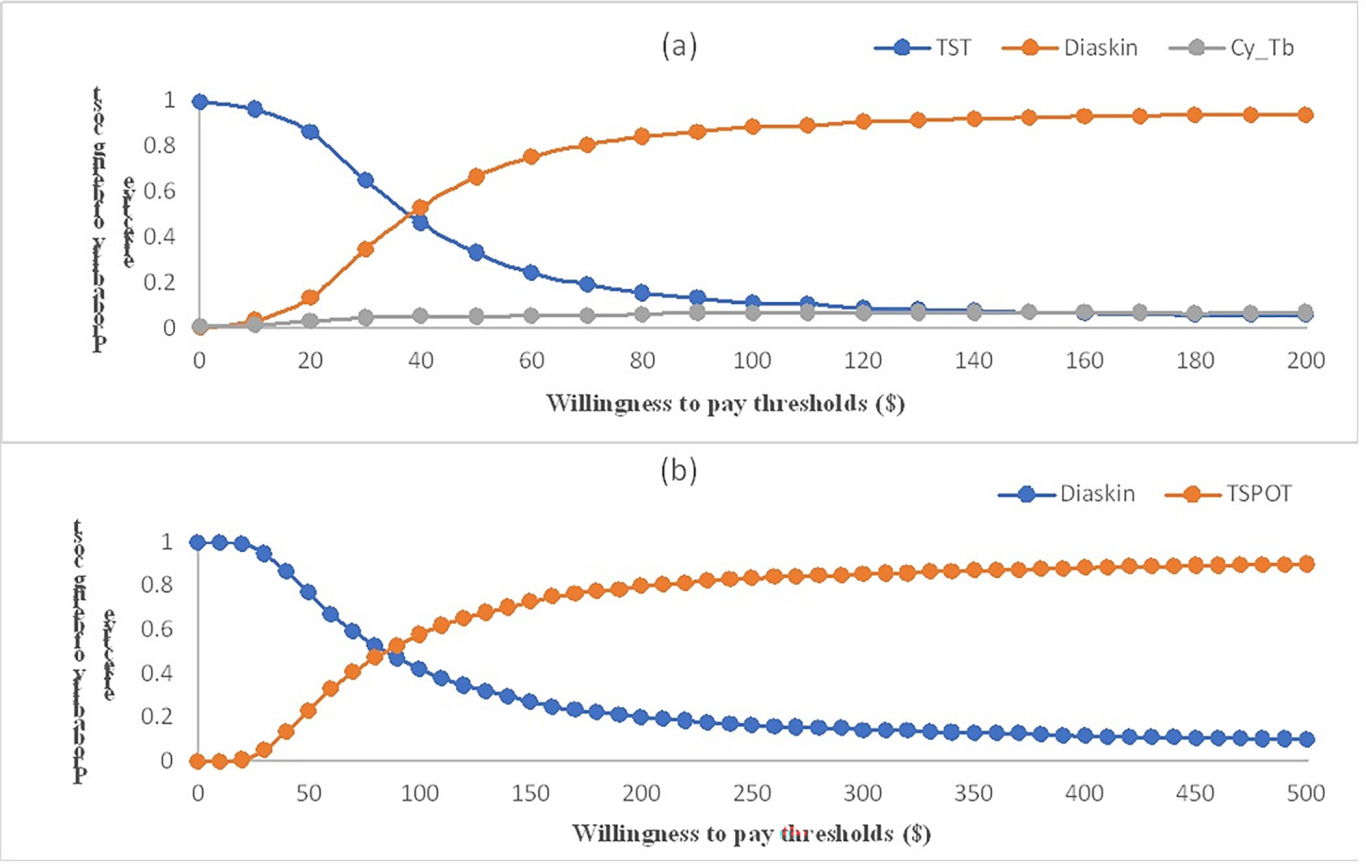
Cost-effectiveness acceptability curve (CEAC) of the different diagnostic strategies for latent tuberculosis infection. CEAC using the net monetary benefit approach with 1000 Monte Carlo simulation showing the probability of (a) all the skin tests, TST-PPD, Diaskintest, and Cy-Tb (b) Diaskintest and TSPOT.TB being cost-effective for a range of willingness-to pay values per correct case diagnosed. The different levels of thresholds (USD, 2021) are shown on X-axis and Y axis represents the represent the probability that each strategy is more cost effective. TST tuberculin skin test.

**Fig 6:**
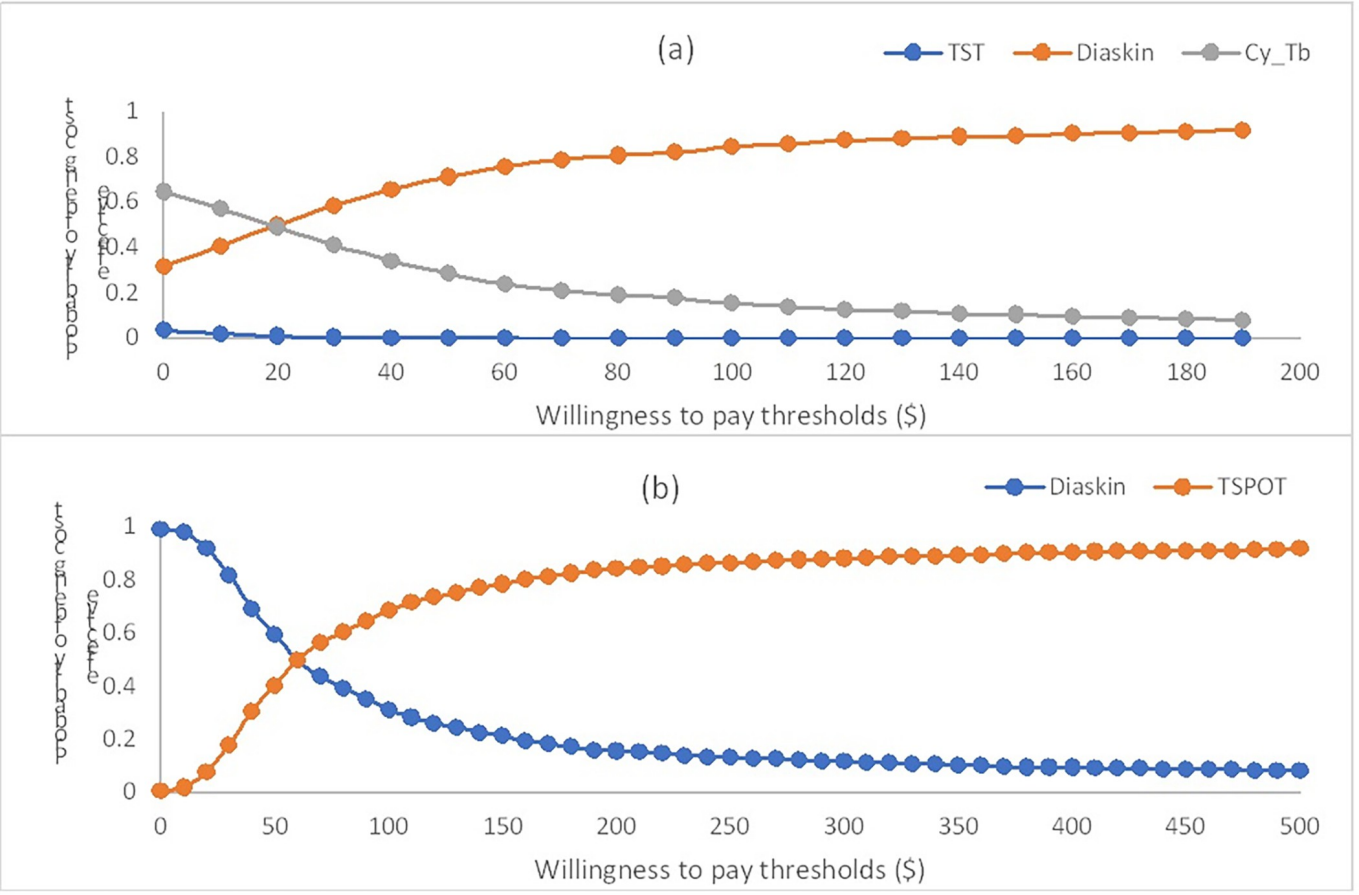
Cost-effectiveness acceptability curve (CEAC) of the different diagnostic strategies for latent tuberculosis infection. CEAC using the net monetary benefit approach with 1000 Monte Carlo simulation showing the probability of (a) all the skin tests, TST-PPD, Diaskintest, and Cy-Tb (b) Diaskintest and TSPOT.TB being cost-effective for a range of willingness-to pay values per correct case diagnosed. The different levels of thresholds (USD, 2021) are shown on X-axis and Y axis represents the represent the probability that each strategy is more cost effective. TST tuberculin skin test.

The findings of the analysis are dependent on parameters obtained from literature and across various time periods. Therefore, sensitivity analyses are conducted to examine how variations in these parameter values affect the estimates of cost-effectiveness. Two types of sensitivity analyses are performed: a one-way sensitivity analysis (Fig 3), which evaluates the impact of uncertainty around each individual parameter, and a Probabilistic Sensitivity analysis (PSA), which assesses the effects of simultaneous uncertainty across multiple parameters. PSA is performed using a Monte Carlo simulation with 1,000 iterations to generate 95% uncertainty range around all estimates to account for parameter uncertainty [24, 25].The uncertainty in diagnostic accuracies and clinical probabilities are assumed to have a beta distribution, while a gamma distribution is assumed for costs. A cost-effectiveness plane was used to illustrate the ICER’s uncertainty (based on the bootstrap samples) (Fig 4). Finally, a Cost-Effectiveness Acceptability Curve (CEAC) is plotted to show the probability of being cost effective for all the diagnostic strategies against a range of willingness to pay thresholds per additional correct case diagnosed in each setting (Figs 5 and 6).

## Results

The results for both settings are shown in Table 2. The first panel displays the results of skin-based tests, whereas the IGRA results are presented in the second panel. The third panel compares the best performing skin test with the best performing IGRA.

**Table 2:** Results.

Strategy	Expected Unit Cost	Incremental Unit Cost	Expected Outcome	Incremental Outcome	ICER	CI (95%)
**India**						
**Skin-based tests**						
TST_PPD	10.280		0.800			
Cy-Tb	11.733	1.453	0.818	0.018	81.98	(71.51, 92.45)
Diaskintest	12.018	1.738	0.843	0.043	40.46	(28.66, 51.84)
**IGRA**						
QFT-GP	23.718		0.950		dominated	
TSPOT.TB	19.966		0.952			
TBF	20.795		0.923		dominated	
**Brazil**						
**Skin-based tests**						
TST_PPD	13.777		0.808		dominated	
Cy-Tb	11.294		0.833			
Diaskintest	11.775	0.482	0.854	0.021	23.47	(4.22, 42.72)
**IGRA**						
QFT-GP	21.265		0.960		dominated	
TSPOT.TB	17.731		0.962			
TBF	21.083		0.933		dominated	
**Skin based test vs Blood based test**						
**Strategy**	**Expected Unit Cost**	**Incremental Unit Cost**	**Expected Outcome**	**Incremental Outcome**	**ICER**	**CI (95%)**
**India**						
Diaskintest	12.018		0.843			
TSPOT.TB	19.966	7.948	0.952	0.109	72.758	(36.16, 108.9)
**Brazil**						
Diaskintest	11.775		0.854			
TSPOT.TB	17.731	5.956	0.962	0.108	54.894	(27.13, 82.65)

Costs provided (inflated) in 2021 USDs. IGRA = interferon-γ release assays, QFT-GP = QuantiFERON-TB Gold Plus, TST-PPD = tuberculin skin test- purified protein derivative, TBF = Standard TB-feron Elisa, CI = confidence interval around the average value of incremental cost effectiveness ratio at 95%, ICER Incremental cost effectiveness ratio

### Skin-based tests

For India, when Diaskintest and Cy-Tb are compared to TST-PPD, both tests. are more effective and have incremental costs of 1.7 USD and 1.5 USD, respectively, per correct TBI case diagnosed. Compared to TST-PPD, Diaskintest and Cy-Tb are cost effective at 40(28.66, 51.84) USD and 82(71.51, 92.45) USD per correct TBI case diagnosed, respectively. Hence, Diaskintest is more cost effective at a lower cost compared to TST-PPD.

In the case of Brazil, the TST-PPD test is dominated when compared to newly developed skin tests as it incurs higher cost and is less effectiveness as compared to Diaskintest and Cy-Tb. When Diaskintest is being compared to Cy-Tb, Diaskintest has an incremental cost of 0.5 USD per correct TBI case diagnosed and is also more effective. Compared to Cy-Tb, Diaskintest is cost effective at 23.5(4.22, 42.72) USD per correct TBI case diagnosed. Thus, Diaskintest is more cost effective at a lower cost compared to TST-PPD in both settings.

### Blood-based tests

For India, when QFT-GP and TSPOT.TB are compared to TBF, both QFT-GP and TBF are dominated, as these tests have higher costs and lower effectiveness than TSPOT.TB. similarly, in case of Brazil, when current IGRAs (QFT-GP and TSPOT.TB) are compared to new blood-based tests (TBF),both QFT-GP and TBF are dominated due to their higher costs and lower effectiveness compared to TSPOT.TB.

### Skin-based tests vs. blood-based tests

Since Diaskintest and TSPOT.TB are the best performing diagnostic tests among the included skin-based and blood-based tests in terms of cost effectiveness, we compared these tests directly (panel 3, Table 2) in both settings. For India, TSPOT.TB has an incremental cost of approximately 8 USD, and demonstrates higher effectiveness compared to Diaskintest with an average ICER of approximately 73(36.16, 108.9)USD. In Brazil, compared to Diaskintest, TSPOT.TB has an incremental cost of approximately 6 USD per correct TBI case diagnosed and has higher effectiveness, resulting in an average ICER of approximately 55(27.13, 82.65)USD.

The tornado plot in Fig 3 demonstrates several key findings. For India, when comparing skin-based tests with the same sensitivity and specificity, the cost of the second visit contributes less to the uncertainty of the ICER compared to factors like loss to follow-up and prevalence. Similarly, when comparing skin-based tests with IGRAs, both prevalence and cost of the second visit contribute less to uncertainty of the ICER. In contrast, for Brazil, the loss to follow-up parameter introduces greater uncertainty in ICER values when skin-based tests are compared with IGRAs. Overall, assuming the consistent sensitivity and specificity, the ICER remains robust across all parameters.

The cost-effectiveness plane in Fig 4 illustrates the incremental cost and incremental effectiveness (i.e. correctly diagnosed cases), with each point on the plane representing a probabilistic simulation. The plot shows that for skin-based tests, the most incremental values fall in the first quadrant, indicating that Diaskintest and Cy-Tb generally have higher costs and higher effectiveness compared to TST. In contrast, for IGRAs, values are distributed across all quadrants, clustering around the origin. This distribution reflects significant uncertainty in the incremental costs and effects of QFT-GP and TBF compared to TSPOT.TB.

The CEAC for various diagnostic strategies in both countries are illustrated in Figs 5 and 6. The probability of Diaskintest being cost-effective, compared to other skin-based tests, increases with increasing willingness-to-pay threshold in both countries.

## Discussion

This study found that Diaskintest is the most cost-effective skin-based test as compared to TST-PPD in both countries, with later being dominated in Brazil. Among the IGRAs, QFT-GP and TBF are dominated in both countries as both have higher cost and lower effectiveness per correct TBI case diagnosed as compared to TSPOT.TB. When compared to Diaskintest, IGRA is costlier and more effective at the same time in both high burden countries. Despite having similar diagnostic accuracy, TSPOT exhibits a higher expected effectiveness compared to Diaskintest may be attributed to two potential factors: the issue of loss to follow-up and the wide variability in the specificity of Diaskintest. Recently, a similar modelling study conducted in three diverse settings—UK, Brazil, and South Africa–similarly found that Diaskintest dominates TST-PPD. While Diaskintest is found to be costlier and gained Quality Adjusted Life Years (QALYs) compared to IGRA, it remained cost effective in South Africa and Brazil. However, in the UK, Diaskintest demonstrated more health gains over both TST and IGRA, and it was found to be cheaper than TST. Nevertheless, it is observed to be more expensive than IGRA, though its health gains implied that the ICER would still be considered cost-effective [17].

More affordable and operationally simpler TB testing options are crucial to increase the diagnostic test availability and acceptability [17, 21]. New skin-based tests offer comparable diagnostic accuracy to IGRAs but at an lower cost [12]. Compared to TST-PPD, these new skin-based tests are more effective in both settings, with Diaskintest showing a lower ICER of 40 USD per correctly diagnosed case. When IGRA and Diaskintest are compared, IGRA demonstrates higher incremental cost and effectiveness per correctly diagnosed case, although its ICER of 74 USD is relatively high. Our findings support the benefits of Diaskintest, aligning with prior economic evaluation literature for TBI diagnostics [17, 26, 27]. Another study demonstrated the incremental health gains for Diaskintest compared with TST-PPD and IGRA. It revealed that Diaskintest is cost-saving at 1375 USD per QALY for a cohort of HIV positive people [21]. However, these findings should be interpreted cautiously, given the limitations and significant heterogeneity in the effectiveness estimates for Diaskintest, as well as the limitations in cost-estimates provided by the manufacturer [17].

This study has several limitations. First, the cost information is obtained from different sources and does not include the transportation cost. As a result, caution is needed when considering scaling up these diagnostic technologies for other developing countries. However, another study included transportation costs across different countries found similar results [17]. Second, the model in this study represents the general population, and due to limited data, it could not account for specific vulnerable groups, immunocompromised patients, drug-resistance, and household contacts. Third, the lack of data prevented conducting sensitivity analysis for different population subgroups limiting the ability to assess the distribution of impact across diverse groups. Lastly, the study could not include the Wantai TB IGRA and C-TST, one of the newly recommended tests by the WHO, due to data limitations.

However, this study also has multiple strengths. To the best of our knowledge, this is the first study to compare the cost effectiveness of a wide range of newly developed skin-based tests and IGRAs, as well as to compare the cost effectiveness of the best performing skin-based test with the best performing IGRA. In addition, this is the first study to model cost effectiveness of diagnostic technologies for TBI specifically for India, the country with the highest TB burden and a producer of TBI test (Cy-Tb).The analysis and findings of the current study are consistent with the previous economic evaluation of latent TBI diagnostics, further validating the results.

## Conclusion

From the health system perspective, Diaskintest and TSPOT.TB emerge as the dominant strategies for skin-based and blood-based TBI testing in India and Brazil, respectively. Diaskintest is cost-saving compared to TSPOT.TB but the latter demonstrates higher effectiveness. This conclusion is based on several assumptions and data limitations. The development of new technologies for TBI testing has increased competition and driven down the costs. However, the decision-makers consider more than just economic analyses; they also consider factors such as ease of implementation, equity, accessibility of supplies, and the potential for scalability. To support the widespread adoption of these new technologies, further research is needed, particularly on equity implications and the budget impact analysis for scalability.

## Supporting information

S1 Codes Visual basic codes for simulations. (DOCX)

## References

[1] Houben R.M. and Dodd P.J., The global burden of latent tuberculosis infection: a re-estimation using mathematical modelling. PLoS medicine, 2016. 13(10): p. e1002152. doi: 10.1371/journal.pmed.1002152 27780211 PMC5079585

[2] World Health Organization, Latent tuberculosis infection: updated and consolidated guidelines for programmatic management. 2018, World Health Organization. 30277688

[3] World Health Organization, WHO consolidated guidelines on tuberculosis: module 1: prevention: tuberculosis preventive treatment. 2020: World Health Organization. 32186832

[4] Hamada Y., Cirillo D.M., Matteelli A., Penn-Nicholson A., Rangaka M.X., and Ruhwald M., Tests for tuberculosis infection: landscape analysis. European Respiratory Journal, 2021. 58(5). doi: 10.1183/13993003.00167-2021 33875495

[5] World Health Organization, Latent tuberculosis infection: updated and consolidated guidelines for programmatic management. 2018: World Health Organization. 30277688

[6] Auguste P., Tsertsvadze A., Pink J., McCarthy N., Sutcliffe P., and Clarke A., Comparing interferon-gamma release assays with tuberculin skin test for identifying latent tuberculosis infection that progresses to active tuberculosis: systematic review and meta-analysis. BMC infectious diseases, 2017. 17(1): p. 1–13. 28274215 10.1186/s12879-017-2301-4 PMC5343308

[7] Pai M., Denkinger C.M., Kik S.V., Rangaka M.X., Zwerling A., Oxlade O., et al., Gamma interferon release assays for detection of Mycobacterium tuberculosis infection. Clinical microbiology reviews, 2014. 27(1): p. 3–20. doi: 10.1128/CMR.00034-13 24396134 PMC3910908

[8] Alsdurf H., Hill P.C., Matteelli A., Getahun H., and Menzies D., The cascade of care in diagnosis and treatment of latent tuberculosis infection: a systematic review and meta-analysis. The Lancet Infectious Diseases, 2016. 16(11): p. 1269–1278. doi: 10.1016/S1473-3099(16)30216-X 27522233

[9] Tebruegge M., Buonsenso D., Brinkmann F., Noguera-Julian A., Pavić I., Arbore A.S., et al., European shortage of purified protein derivative and its impact on tuberculosis screening practices. The International Journal of Tuberculosis and Lung Disease, 2016. 20(10): p. 1293–1299. doi: 10.5588/ijtld.15.0975 27725037

[10] Loureiro R.B., Maciel E.L.N., Caetano R., Peres R.L., Fregona G., Golub J.E., et al., Cost-effectiveness of QuantiFERON-TB Gold In-Tube versus tuberculin skin test for diagnosis and treatment of Latent Tuberculosis Infection in primary health care workers in Brazil. PloS one, 2019. 14(11): p. e0225197. 31725786 10.1371/journal.pone.0225197 PMC6855475

[11] Marx F.M., Hauer B., Menzies N.A., Haas W., and Perumal N., Targeting screening and treatment for latent tuberculosis infection towards asylum seekers from high-incidence countries–a model-based cost-effectiveness analysis. BMC Public Health, 2021. 21(1): p. 1–16. 34836526 10.1186/s12889-021-12142-4 PMC8622109

[12] Krutikov M., Faust L., Nikolayevskyy V., Hamada Y., Gupta R.K., Cirillo D., et al., The diagnostic performance of novel skin-based in-vivo tests for tuberculosis infection compared with purified protein derivative tuberculin skin tests and blood-based in vitro interferon-γ release assays: a systematic review and meta-analysis. The Lancet Infectious Diseases, 2021. 10.1016/S1473-3099(21)00261-9 34606768

[13] World Health Organization, Rapid communication: TB antigen-based skin tests for the diagnosis of TB infection. 2022, World Health Organization.

[14] Badaro R., Machado B., Duthie M.S., Araujo-Neto C., Pedral-Sampaio D., Nakatani M., et al., The single recombinant M. tuberculosis protein DPPD provides enhanced performance of skin testing among HIV-infected tuberculosis patients. Amb Express, 2020. 10(1): p. 1–8. 10.1186/s13568-020-01068-6 PMC7394993 32737693

[15] Edgar Ortiz-Brizuela L.A., Sophie Lachapelle-Chisholm, Zhiyi, Tania Mukherjee, Michele Miedy, and Dick Menzies, McGill University., Assessing the diagnostic performance of five new blood-based interferon-gamma release assays: a systematic review and meta-analysis. 2022.

[16] World Health Organization, Use of alternative interferon-gamma release assays for the diagnosis of TB infection: WHO policy statement. 2022.

[17] Goscé L., Allel K., Hamada Y., Korobitsyn A., Ismail N., Bashir S., et al., Economic evaluation of novel Mycobacterium tuberculosis specific antigen-based skin tests for detection of TB infection: A modelling study. PLOS Global Public Health, 2023. 3(12): p. e0002573. doi: 10.1371/journal.pgph.0002573 38117825 PMC10732392

[18] World Health Organization, Global tuberculosis report 2021.

[19] GBD, Global, regional, and national incidence, prevalence, and years lived with disability for 354 diseases and injuries for 195 countries and territories, 1990–2017: a systematic analysis for the Global Burden of Disease Study 2017. Lancet, 2018. 392(10159): p. 1789–1858. doi: 10.1016/S0140-6736(18)32279-7 30496104 PMC6227754

[20] Roberts M., Russell L.B., Paltiel A.D., Chambers M., McEwan P., and Krahn M., Conceptualizing a model: a report of the ISPOR-SMDM modeling good research practices task force–2. Medical Decision Making, 2012. 32(5): p. 678–689. doi: 10.1177/0272989X12454941 22990083

[21] Steffen R.E., Pinto M., Kritski A., and Trajman A., Cost-effectiveness of newer technologies for the diagnosis of Mycobacterium tuberculosis infection in Brazilian people living with HIV. Scientific reports, 2020. 10(1): p. 1–12. 33311520 10.1038/s41598-020-78737-w PMC7733491

[22] Value TB, Value TB Dataverse, https://dataverse.harvard.edu/dataverse/Value-TB. 2021.

[23] Steffen R.E., Caetano R., Pinto M., Chaves D., Ferrari R., Bastos M., et al., Cost-effectiveness of Quantiferon®-TB Gold-in-Tube versus tuberculin skin testing for contact screening and treatment of latent tuberculosis infection in Brazil. PloS one, 2013. 8(4): p. e59546. 23593145 10.1371/journal.pone.0059546 PMC3617186

[24] Drummond M.F., Sculpher M.J., Claxton K., Stoddart G.L., and Torrance G.W., Methods for the economic evaluation of health care programmes. 2015: Oxford university press.

[25] Gray A.M., Clarke P.M., Wolstenholme J.L., and Wordsworth S., Applied methods of cost-effectiveness analysis in healthcare. Vol. 3. 2011: Oxford University Press.

[26] Alsdurf H., Empringham B., Miller C., and Zwerling A., Tuberculosis screening costs and cost-effectiveness in high-risk groups: a systematic review. BMC Infectious Diseases, 2021. 21: p. 1–22. 34496804 10.1186/s12879-021-06633-3 PMC8425319

[27] Tasillo A., Salomon J.A., Trikalinos T.A., Horsburgh C.R., Marks S.M., and Linas B.P., Cost-effectiveness of testing and treatment for latent tuberculosis infection in residents born outside the United States with and without medical comorbidities in a simulation model. JAMA internal medicine, 2017. 177(12): p. 1755–1764. doi: 10.1001/jamainternmed.2017.3941 29049814 PMC5808933

