# Supplementary material for: Cost-effectiveness of diagnostic technologies for mycobacterium tuberculosis infection in India and Brazil
Source: PLOS Glob Public Health. 2024 Nov 13;4(11):e0003638. doi: 10.1371/journal.pgph.0003638 (PMC11559971; doi:10.1371/journal.pgph.0003638)
Supplement: S1 Codes — (DOCX) [file pgph.0003638.s001.docx]

**S1 Codes: Visual Basic Codes for Simulations**

**Sub simulations()**

**'**

**' simulations Macro**

**'**

**Sheets("PSA").Select**

**Range("B1").Select**

**ActiveCell.FormulaR1C1 = "1"**

**Sheets("simulations (2)").Select**

**Application.DisplayStatusBar = True**

**Index = 0**

**Trials = 1000**

**Do**

**Range("B5:Y5").Select**

**Selection.Copy**

**Range("B7:Y7").Select**

**ActiveCell.Offset(Index, 0).Range("A1").Select**

**Selection.PasteSpecial Paste:=xlPasteValues, Operation:=xlNone, SkipBlanks _**

**:=False, Transpose:=False**

**Index = Index + 1**

**Loop While Index < Trials**

**Application.DisplayStatusBar = False**

**Sheets("PSA").Select**

**Range("B1").Select**

**ActiveCell.FormulaR1C1 = "0"**

**Sheets("simulations (2)").Select**

**Range("A2").Select**

**End Sub**
